# VIP-2 with modulated current: pathfinder for enhanced Pauli exclusion principle violation studies

**DOI:** 10.1140/epjc/s10052-024-12599-8

**Published:** 2024-03-01

**Authors:** Alessio Porcelli, Massimiliano Bazzi, Nicola Bortolotti, Mario Bragadireanu, Michael Cargnelli, Alberto Clozza, Luca De Paolis, Raffaele Del Grande, Carlo Guaraldo, Mihail Iliescu, Matthias Laubenstein, Simone Manti, Johann Marton, Marco Miliucci, Fabrizio Napolitano, Kristian Piscicchia, Alessandro Scordo, Francesco Sgaramella, Diana Laura Sirghi, Florin Sirghi, Oton Vazquez Doce, Johann Zmeskal, Catalina Curceanu

**Affiliations:** 1 Laboratori Nazionali di Frascati, Istituto Nazionale di Fisica Nucleare, Via Enrico Fermi 54, 00044 Frascati, Italy; 2 Centro Ricerche Enrico Fermi, Museo Storico della Fisica e Centro Studi e Ricerche “Enrico Fermi”, Via Panisperna 89 A, 00184 Rome, Italy; 3 Faculty of Physics, Astronomy and Applied Computer Science, Jagiellonian University, ul. prof. Stanisława Łojasiewicza 11, 30-348 Kraków, Poland; 4 Physics Department, “Sapienza” University of Rome, Piazzale Aldo Moro 5, 00185 Rome, Italy; 5 IFIN-HH, Institutul National pentru Fizica si Inginerie Nucleara Horia Hulubei, 30 Reactorului, 077125 Măgurele, Romania; 6 Stefan-Meyer-Institute for Subatomic Physics, Austrian Academy of Science, Wiesingerstraße 4, 1010 Wien, Austria; 7 Excellence Cluster Universe, Technische Universität München, Arcisstraße 21, 80333 Münich, Germany; 8 Laboratori Nazionali del Gran Sasso, Istituto Nazionale di Fisica Nucleare, Via G. Acitelli 22, 67100 Assergi, Italy; 9 Present Address: Agenzia Spaziale Italiana, Via del Politecnico snc, 00133 Rome, Italy

## Abstract

Fermions are subject to the Pauli Exclusion Principle (PEP), which is grounded on the spin-statistics theorem and, hence, related to the very same structure of the underlying symmetries. The VIP-2 (VIolation of Pauli exclusion principle - 2) experiment has been performing extreme sensitivity tests of the PEP, up to its current and final configuration, exploiting several experimental setups designed to study different theoretical models of PEP violation, looking for a faint signal of physics Beyond the Standard Model.A current is introduced in the copper target to bring new electrons into the system and, hence, fulfill the requirements of the Messiah-Greenberg Super-Selection rule. The searched spin-statistics violating signal corresponds to X-rays emitted when the new electrons perform atomic transitions to the already filled fundamental level of copper. This work analyzes the set of the VIP-2 data corresponding to a test run of 68 days in a current modulated regime alternating no current with current data-taking in short periods (50 s each), instead the usual alternating months-long data-taking of each of these two phases. We propose an analysis method to improve the experiment’s sensitivity: a spectral analysis constraint with the Discrete Fourier Transformation of the data. Compared to the spectrum-only analysis, about a factor of 1.5 of improvement to the limit for the probability of PEP violation for electrons was obtained.

## Pauli exclusion principle violation

The Pauli Exclusion Principle (PEP) is a consequence of the Spin Statistic Theorem [1] and is a pillar of quantum mechanics, responsible for plenty of physical phenomena, among which the stability of matter [2]. However, an intuitive explanation for the distinction between fermionic and bosonic statistics is still needed [3]. VIP-2 (VIolation of Pauli exclusion principle - 2) is performing extreme sensitivity tests of the PEP for electrons, searching for possible signal of new physics in the context of several theoretical scenarios.

Proposed reasons for a PEP Violation (PEPV) occurrence are either related to the nature of the particles, for example, the paronic field [4–6], or theories Beyond Standard Model, for instance, Non-Commutative Quantum Gravity (NCQG) models as per $\theta$-Poincaré [7]. Both predict a certain degree of PEPV.

The first class of models follows the Messiah-Greenberg Super-Selection (MGSS) rule [8], for which transitions among states with different symmetries are forbidden. Therefore, in this context, an experimental search of PEPV needs to introduce new fermions in a pre-existing system of identical fermions and check for the newly formed symmetry state. This class of experiments goes under the name of “Open Systems” tests.

The second class of theories is not constrained by MGSS. One does not need an injection of new particles since the ones from the system might violate the PEP spontaneously. Such a class of experiments are called “Closed Systems” experiments [9, 10].

**Fig. 1:**
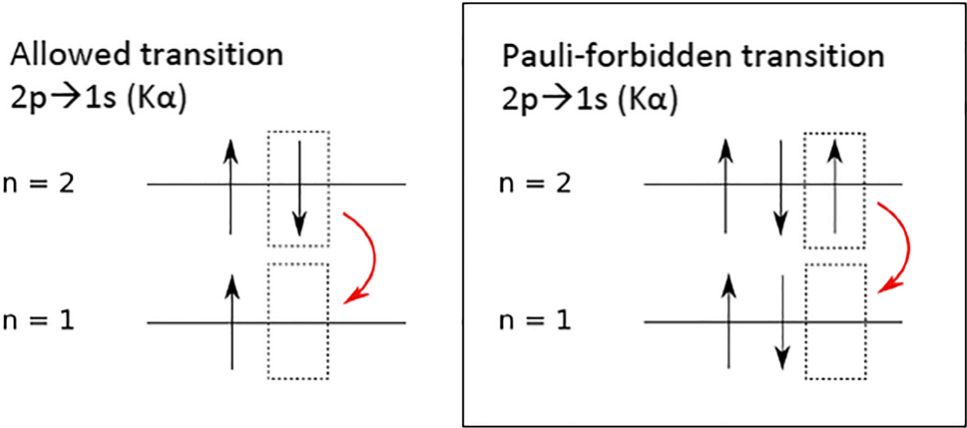
Schematic of PEP-allowed and PEP-violating K${}_\alpha$ transition, respectively, on the left and the right. Reproduced from [12]

VIP-2 tests PEPV in an Open System, detecting X-rays emitted in a copper target circulated by a current. The experimental principle follows the pioneering work performed in 1988 by Ramberg and Snow [11]. X-rays from the K${}_\alpha$ transition (from 2*p* to 1*s*) are emitted with the standard energy of 8047.78 eV for copper K${}_{\alpha 1}$ and 8027.83 eV for K${}_{\alpha 2}$. As new electrons are injected through the current in the copper target, their capture in atomic orbitals is a test for the PEPV. K${}_\alpha$ emissions originating from Pauli-forbidden atomic transitions (Fig. 1) could be observed in a low-background environment with high-precision X-ray spectroscopy. The energy of the PEPV K${}_\alpha$ transition in copper is expected to be shifted down by about 300 eV (7746.73 eV) due to the additional electron shielding from the fully occupied ground state of the atom.

Ignatiev and Kuzmin parametrized PEP violation for electrons in terms of a two-level Fermi oscillator, with $\beta$ the amplitude for a classically forbidden third-level state [5]. The resulting probability of a third electron occupying the 1*s* state is $\beta ^2/2$. For the VIP-2 experiment, the expected number $N_x$ of PEPV events is

1 $$\begin{aligned} N_x \simeq \frac{\beta ^2}{2}\cdot N_\text {new}\cdot \frac{N_\text {int}}{10}\cdot \text {efficiency}. \end{aligned}$$

$N_\text {new}$ is the total number of “new” electrons injected into the system given by the current intensity and data acquisition time. $N_\text {int}$ is the number of electron-atom encounters. The factor 1/10 is an estimation of the capture probability into the 2*p* state as shown in [13]. Finally, the “efficiency” considers the solid angle covered by the detector, the X-ray absorption in the target strip, and the detector efficiency. The experiment of Ramberg and Snow set an upper limit for the PEPV probability for electrons of $\beta ^2/2 < 1.7 \times 10^{-26}$. The VIP experiment, the precursor of VIP-2, improved this limit by about two orders of magnitude [14]; the studies in [13] by one.

In the past, the number of electron-atom encounters $N_\text {int}$ was estimated with a “scattering” model: an electron encounters an atom at every radiation length inside the target. This model is conservative and underestimates the number of encounters, since the scattering is due to phonons and lattice irregularities. Recently, a new model was developed using a more realistic diffusion random walk model: the “close encounters” [15]. The results of this analysis are presented in the context of both approaches.

In this work, we present a new method of data-taking and analysis to improve the current measurements of the $\beta ^2/2$. The approach is driven by the work in [16] using a semi-analytical Monte Carlo simulation. Events are simulated with a modulated current with a regular period that systematically introduces PEPV events. Their Fourier Transformation shows a clear harmonic at the current frequency. We applied this modulated current idea in a test run of the VIP-2 experiment, switching the current on and off with a regular periodicity instead of the usual alternating months-long data-taking for each case. The analysis adapts the concept to our experimental data-taking and the real case, where signal events are, if any, rare with respect to systematic.

## The VIP-2 experiment

**Fig. 2:**
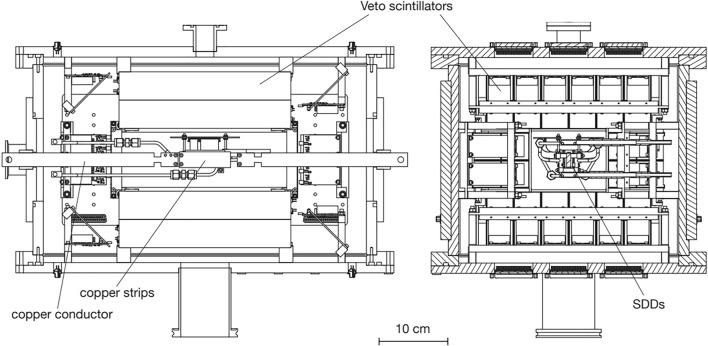
Schematic of the VIP-2 setup (from [17]): in evidence inside the vacuum chamber, the targets (copper strips), the copper conductor used to inject current, and the SDDs

The VIP-2 apparatus is sited in the underground Gran Sasso National Laboratory (LNGS), in Italy, beneath about 1400 m of rock, shielding it from the secondary Cosmic Rays ($\mu$-flux reduced by a factor of about $10^{6}$). It comprises a vacuum chamber containing 32 Silicon Drift Detectors (SDDs) and two parallel copper strips as a target (see Fig. 2 for the schematics). The vacuum chamber is evacuated at a pressure below $10^{-5}$ mbar, allowing the SDDs to be cooled down safely. Moreover, an external shielding was installed surrounding the VIP-2 vacuum chamber to further reduce the natural background produced by the residual radioactivity of the rocks inside the cavern of the LNGS. This outer shielding consists of an inner layer of copper bricks and an exterior layer of lead blocks. A PT-100 sensor is installed on the external surface of the vacuum chamber to monitor the temperature within the shielding, kept fixed at 24 ${}^\circ$C through an air cooling system. We reported the preliminary analysis performed before and during the completion of the external shielding (208 days of data) in [18].

The two copper strips have dimensions of 76 mm long, 20 mm high, and 25 $\upmu$m thick each. During the reported data-taking period, a Direct Current of 180 A was circulated (*wc*) alternated to periods without current (*woc*).

The 32 SDDs are installed in the apparatus for the X-ray spectroscopy. They are organized in 4 arrays $2\times 4$, arranged in pairs to form a matrix $4\times 4$ per outer side of a copper strip. Each SDD is 450 $\upmu$m thick and has an active area of 0.64 cm$^2$ for a total of 5.12 cm$^2$ per array. A cryocooler keeps SDDs to a temperature of 150 K. Six PT-100 sensors are installed inside the vacuum chamber to monitor each SDD array’s and target strips’ temperatures.

A cooling water circuit is installed on the copper strips to avoid a high rise in the target temperature when the current circulates, which could affect the high-quality performance of SDDs. The copper target is kept at a temperature of 20–25 ${}^\circ$C. In these working conditions, SDDs provide an energy resolution for X-rays of about 190 eV Full-Width Half Maximum (FWHM) at 8 keV with a detection efficiency of more than 99%. Such energy resolution allows to disentangle the PEPV transitions from the standard ones in the energy spectrum. SDD working principles and schemes are detailed in [17].

Finally, to perform *in situ* SDD calibrations, we placed a Fe-55 source below the target covered by a 25 $\upmu$m thick Titanium foil. This way, the K$_\alpha$ and K$_\beta$ lines emitted by Mn and Ti are used for the SDD calibration. The calibration is executed in batches of approximately ten days for each SDD detector, translating their ADC counts into Energy (in eV).

The final configuration of the VIP-2 experiment, with the complete external shielding, was installed in LNGS in April 2019 and, since then, is in data taking.

### The modulated current data taking

The standard VIP-2 Open System data-taking campaign consists of weeks-long phases with current (*wc*) alternated with similarly long phases without current (*woc*). The latter is a background reference (see Sect. 3).

We performed a test run acquiring 68 days of data between October and December 2020. In this run, we introduced the *modulated current data-taking*, for which the *wc*-*woc* alternation is automatized with a fixed period of 100 s: 50 s of *wc* phase, 50 s of *woc*, with a time precision of 1 s.

## Spectral analysis

The spectrum region of interest for studying the PEPV is selected as from 7270 to 8300 eV. It includes the copper K$_\alpha$ lines ($E^\textrm{Cu}_{K\alpha 1} = 8047.78$ eV and $E^\textrm{Cu}_{K\alpha 2} = 8027.83$ eV) with a small contamination from nickel K$_\alpha$ line ($E^\textrm{Ni}_{K\alpha 1} = 7478.15$ eV). This contamination is due to the ceramic support of the SDD arrays. Only Ni K$_{\alpha 1}$ can be distinguished due to low statistics. The copper has a relative ratio between the K$_{\alpha 2}$ and K$_{\alpha 1}$ amplitudes of 0.51, as well known from the literature [19]. Moreover, we consider $E^\textrm{Cu}_{K\alpha 2} = E^\textrm{Cu}_{K\alpha 1}-19.95$ eV to reduce the number of variables since their energy difference is precisely measured.

As reported in previous publications [18, 20], the VIP-2 standard approach is a Bayesian analysis of a combined spectra Likelihood $\mathcal {L} = \mathcal {L}^{woc}\cdot \mathcal {L}^{wc}$ using the Markovian Chain Monte Carlo techniques [21] (Metropolis-Hasting algorithm [22]). Both the Likelihood factors are the product of bin-by-bin Poissonian distributions $\mathcal {P}(n\,|\,\lambda )$, where the counts of the *i*-th bin are the data $\mathcal {D}_i$ and a function of the bin energy $\mathcal {F}(E_i)$ is the model.

One Likelihood factor is modeled for the *woc* phase as follows:

2 $$\begin{aligned}&\mathcal {L}^{woc}(\varvec{\mathcal {D}}^{woc},\varvec{\mathcal {F}}^{woc}) \nonumber \\&\quad =\prod _i \mathcal {P}(\mathcal {D}^{woc}_i \,|\, \mathcal {F}^{woc}(E_i\,|\,\varvec{\theta })) \end{aligned}$$

3 $$\begin{aligned} \mathcal {F}&^{woc}(E_i\,|\,\varvec{\theta }) \nonumber \\&\quad =a\cdot (E_i-E^\textrm{Cu}_\textrm{PEPV}) + b\,\nonumber \\&\qquad +\left\{ A^\textrm{Ni}\cdot \mathcal {N}(E_i-E^\textrm{Ni}_{K\alpha },\sigma ^\textrm{Ni})\right. \nonumber \\&\qquad +\,A^\textrm{Cu}\cdot \left[ \mathcal {N}(E_i-E^\textrm{Cu}_{K\alpha 1},\sigma ^\textrm{Cu})\right. \nonumber \\&\qquad +\left. \left. 0.51\cdot \mathcal {N}(E_i-E^\textrm{Cu}_{K\alpha 1}+19.95,\sigma ^\textrm{Cu}) \right] \right\} w_\textrm{bin}\,. \end{aligned}$$

$\mathcal {N}(E,\sigma )$ are normal distributions with the centroid centered around the expected energy peaks and a standard deviation given by the detector resolution (slightly different for different energies). $w_\textrm{bin}$ is the bin width (10 eV) used as a factor, so thus $A^\textrm{Ni}$ and $A^\textrm{Cu}$ are expressed as a total number of events (respectively for the copper or the nickel emissions). The background is described by a linear function with a slope *a*. The function is centered on the PEPV expected energy ($E^\textrm{Cu}_\textrm{PEPV} = 7746.73$ eV); in this way, the interpretation of the *b* parameter is the background counts at the signal energy. With $\varvec{\theta }$, we express the vector of all parameters, later discussed.

The second Likelihood factor is modeled for the phase with current *wc*, which is the same as $\mathcal {F}^{woc}$ but with one more normal distribution to describe the PEPV signal with a total number of events *S*:

4 $$\begin{aligned}&\mathcal {L}^{wc}(\varvec{\mathcal {D}}^{wc},\varvec{\mathcal {F}}^{wc}) \nonumber \\&\quad =\prod _i \mathcal {P}(\mathcal {D}^{wc}_i \,|\, \mathcal {F}^{wc}(E_i\,|\,\varvec{\theta })) \end{aligned}$$

5 $$\begin{aligned}&\mathcal {F}^{wc}(E_i\,|\,\varvec{\theta }) \nonumber \\&\quad =a\cdot (E_i-E^\textrm{Cu}_\textrm{PEPV}) + b\, \nonumber \\&\qquad +\left\{ A^\textrm{Ni}\cdot \mathcal {N}(E_i-E^\textrm{Ni}_{K\alpha },\sigma ^\textrm{Ni})\right. \nonumber \\&\qquad +\,A^\textrm{Cu}\cdot \left[ \mathcal {N}(E_i-E^\textrm{Cu}_{K\alpha 1},\sigma ^\textrm{Cu})\right. \nonumber \\&\qquad +\left. 0.51\cdot \mathcal {N}(E_i-E^\textrm{Cu}_{K\alpha 1}+ 19.95,\sigma ^\textrm{Cu}) \right] \nonumber \\&\qquad +\left. S\cdot \mathcal {N}(E_i-E^\textrm{Cu}_\textrm{PEPV},\sigma ^\textrm{Cu})\right\} w_\textrm{bin}\,. \end{aligned}$$

The PEPV signal normal distribution is centered around $E^\textrm{Cu}_\textrm{PEPV}$ with the copper standard deviation; therefore, the same $\sigma ^\textrm{Cu}$ is used.

Using the Bayesian inference, the posterior is given by equation 6:

6 $$\begin{aligned} p(\varvec{\theta }\,|\,\varvec{\mathcal {D}}^{woc},\varvec{\mathcal {D}}^{wc}) = \frac{\mathcal {L}(\varvec{\mathcal {D}}^{woc},\varvec{\mathcal {D}}^{wc}\,|\,\varvec{\theta })\cdot p(\varvec{\theta }) }{\int \textrm{d}\varvec{\theta }\, \mathcal {L}(\varvec{\mathcal {D}}^{woc},\varvec{\mathcal {D}}^{wc}\,|\,\varvec{\theta })\cdot p(\varvec{\theta }) }\,, \end{aligned}$$

where the Likelihood is expressed as

7 $$\begin{aligned} \mathcal {L}(\varvec{\mathcal {D}}^{woc},\varvec{\mathcal {D}}^{wc}\,|\,\varvec{\theta })&= \mathcal {L}^{woc}(\varvec{\mathcal {D}}^{woc},\varvec{\mathcal {F}}^{woc}(\varvec{\theta }\,|\,\varvec{E}))\,\cdot \nonumber \\&\quad \cdot \mathcal {L}^{wc}(\varvec{\mathcal {D}}^{wc},\varvec{\mathcal {F}}^{wc}(\varvec{\theta }\,|\,\varvec{E})) \end{aligned}$$

and $p(\varvec{\theta })$ indicates the product of the priors probability density functions of the parameters $\varvec{\theta }$. They are the number of events $A^\textrm{Ni}$ and $A^\textrm{Cu}$, the slope *a*, the background count at the PEPV Energy *b*, and the signal count *S*, all with uniformly distributed priors. Because the two data sets might differ, all parameters in each model are considered independent, doubling all, except for *S*, for a total of nine parameters. Considering the systematic uncertainties of the calibration, $E^\textrm{Cu}_{K\alpha 1}$ and $E^\textrm{Ni}_{K\alpha }$ become two more free parameters. Their priors are expressed as normal distributions centered in their known values and with a standard deviation of the calibration uncertainty (2 eV). $E^\textrm{Cu}_\textrm{PEPV}$ is considered fixed. Furthermore, we included $\sigma ^\textrm{Cu}$ and $\sigma ^\textrm{Ni}$ as two extra free parameters uniformly distributed to consider the systematic uncertainties of the detector resolution. The resolution of the SDDs is shared by the two data sets. As a final systematic uncertainty, we considered the 1 s precision uncertainty of the data timestamp as a scaling factor to the $\mathcal {F}^{woc}$: $\mathcal {L}^{woc} = \prod _i \mathcal {P}(\mathcal {D}^{woc}_i \,|\, \textrm{scale}\cdot \mathcal {F}^{woc}(E_i\,|\,\varvec{\theta }))$. The scale factor has a prior normally distributed, centered to 1 (the ratio of the time of data acquisition with and without current is exactly 1) with a standard deviation of 1 s over 34 days of a single phase data acquisition time. Counting all, the total number of free parameters $\varvec{\theta }$ is fourteen.

**Fig. 3:**
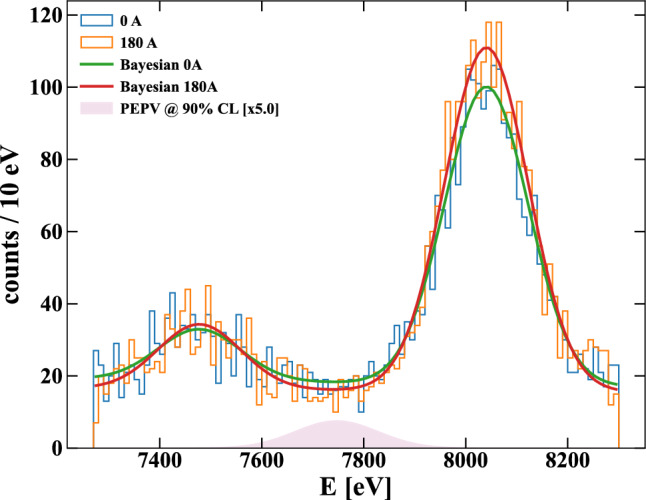
Energy spectra of the VIP-2 calibrated data without (blue) and with (orange) the current. Their Bayesian optimizations are shown (green and red, respectively); the signal component distribution inside the upper limit at 90% of C.L. is shown in pink, magnified by a factor of 5

The result of the Bayesian analysis is shown in Fig. 3 as green and red lines for the without current (blue distribution) and with current (orange distribution), respectively. Since *S* is the parameter of interest, we also show its distribution in pink, magnified by a factor of 5 for visibility reasons. No significant signal is found: the shown shaded area represents the signal distribution inside 90% C.L. of *S* counts. Thus, the signal upper limit obtained is $\bar{S}_\text {spec} = 16.11$ events at 90% of C.L., corresponding to a limit on the PEPV probability of

8 $$\begin{aligned} \left. \frac{\beta ^2}{2}\;\right| _\text {spec} < 1.25\cdot 10^{-30}&\qquad \text {(scattering)} \end{aligned}$$

9 $$\begin{aligned} \left. \frac{\beta ^2}{2}\;\right| _\text {spec} < 9.94\cdot 10^{-43}&\qquad \text {(close encounters)}\,. \end{aligned}$$

## Modulated current analysis

**Fig. 4:**
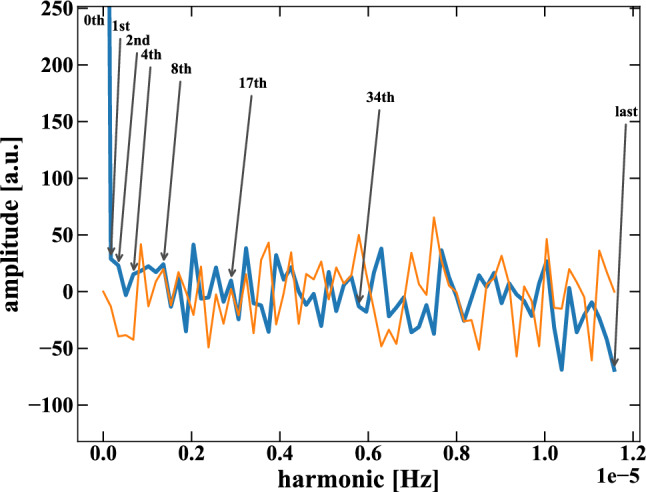
Real (blue) and Imaginary (orange) parts of the DFT, using the data after the elaboration described in Sect. 4.1. The 0-th, last, and other harmonic of interest (“central harmonics”) are shown. The y-axis upper limit is cut at 250 to evidence the fluctuations of all the harmonics (the amplitude of the 0-th harmonic is about 1200)

We consider every phase, with and without current, as a bin and its number of events as the bin content (the first bin is a *wc*) to build the data structure with a period *T*, and we apply the Discrete Fourier Transform (DFT) [23] (the algorithm used is the Fast Fourier Transform). The data *D* comprises the background counts *B* in all bins and the signal events *S* only in the *wc* ones.

The DFT splits the binning into different multiples of *T*, assigning an amplitude (complex number) to each of those harmonics. In Fig. 4, the harmonic amplitudes for the Real (in blue) and Imaginary (orange) parts of the DFT are shown (the y-axis upper limit is cut at 250). The harmonic of interest where the signal presence can appear is in the “last” one (i.e., the 68th harmonic in this case), representing the period *T*, i.e., the switch between current and without current. The Imaginary amplitude of the last bin is 0 by construction; therefore, all Imaginary parts are of no interest. Other harmonics of interest split the period with an equal content of *wc* and *woc* bins: in this case, they are the 1st, 2nd, 4th, 8th, 17th, and 34th, now referred to as “central harmonics.” The 0-th harmonic is, trivially, the total sum of all the events; therefore, it is not of interest.

The last bin represents the difference of events between all *wc* and all *woc* parts, similar to a spectrum subtraction analysis. The limit of a spectrum subtraction is the strong presence of uncertainties due to the Poissonian fluctuations from both spectra. However, if one can infer about the Poissonian behavior of the data set, the residue is the signal. In other words, the last bin is the Poissonian distribution of the background *B* shifted up (because the first bin of the data structure is *wc*) by the signal *S*. The information about the Poissonian behavior of the data is inside the central harmonics, since they have an equal content of *wc* and *woc* bins. Therefore, all their amplitudes are expected to be Normally distributed around a common mean and a variance. Thoroughly understanding this behavior lets us constrain the signal residue in the last bin. In Sect. 4.2, their behavior and the possible small dependence on the signal presence are studied data-driven.

### Modulated current data

The structure of the modulated current data must be regular: all concatenated, no dead time, exact period alternating *wc* and *woc* where the first time bin (i.e., the first 50 s) is a *wc* while the last one is a *woc*. To analyze these data, we restrict to the same energy range of the spectral analysis: from 7270 to 8300 eV.

We identify a Region of Interest (ROI) as a 150 eV neighborhood (left and right) of the PEPV energy $E^\textrm{Cu}_\textrm{PEPV} = 7746.73$ eV: from 7596.73 to 7896.73 eV. This 300 eV wide energy region is chosen, so thus, exceeding events might belong to about 95% of the PEPV distribution; in other words, this is a signal-enriched region. The remaining signal-depleted part is the Background (BKG) region: $(7270\le E[\textrm{eV}]<7596.73)\cup (7896.73\le E[\textrm{eV}]<8300)$.

In the VIP-2 case, the ROI is a region where 50 s of data-taking is too short to have enough rate per time bin. Therefore, to avoid bias toward the 0 events case, we grouped the *wc* and *woc* bins to have a period large enough for having no empty bins. The period of the regrouped data set is $T=24$ hours (shown in Fig. 4): 12 h *wc* and 12 h *woc*. The regrouping is possible without losing information or generality from the DFT perspective.

### Behaviors of the DFT central harmonics

**Fig. 5:**
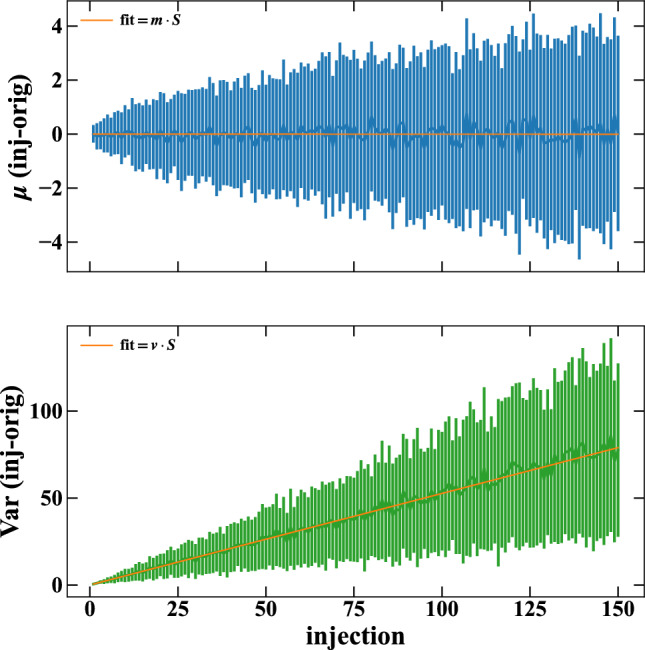
Average (top, in blue) and Variance (bottom, in green) of the difference between the synthetic (synth) and the original (orig) data set as a function of signal hypotheses. Vertical bars correspond to each hypothesis’s total spread of the generated synthetic data. The linear fit is shown in orange. 100 synthetic data sets from the ROI subset were generated for each $S>0$ hypothesis

The data set is an ensemble of $D=B+S$ events, where *B* has an unknown behavior. Since we know *D*, we can study it empirically as a function of hypothetical *S* to describe it. The goal is to understand the DFT central harmonic dependencies from the signal presence even in these harmonics. We assume a Normal distribution with a mean $\mu$ and a variance Var to describe fluctuations. Therefore, we study how these parameters change under different *S* hypotheses.

We build a synthetic data set from the data *D* by subtracting random events from the *wc* bins as a signal *S* hypothesis. The resulting central harmonics are the DFT of a possible *B*. The mean and variance of the differences from the original data set will show their behavior as a function of *S* without any assumption on *D* and *B*.

We generated 100 synthetic data sets for each signal hypothesis and compared it with the original data set (synthetic − original). In Fig. 5, the $\mu$ and Var of this difference are depicted, top (in blue) and bottom (in green), respectively; the vertical bars correspond to the total spread of the generated synthetic data for each hypothesis. Since $S=0$ corresponds to the no subtraction, it is the original case; therefore, only $S>0$ hypotheses are shown. A linear fit is performed (orange lines), with the slope as a free parameter and the intercept fixed to 0 (trivially, no variation of the original case from itself).

The fit result for the $\mu$ has a slope of 0. It shows an independence from the signal. Instead, the result from the Var highlights a linear dependence from possible signals. Therefore, we can build a data-driven model for the Variance as function of *S*:

10 $$\begin{aligned} \textrm{Var} = V_0 + v\cdot S, \end{aligned}$$

where $V_0$ is the (unknown) baseline Variance, and *v* is the slope (about 0.5 in the fit) of the linear dependence from the signal *S*.

**Fig. 6:**
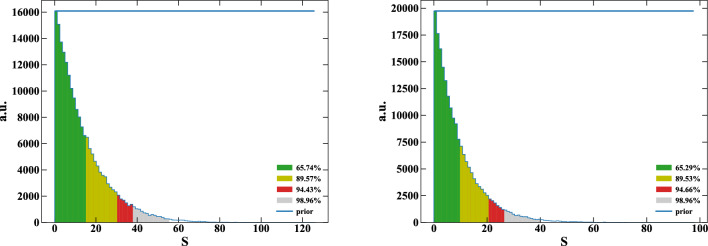
Marginalized posterior distribution of *S* using only the spectral analysis as per Sect. 3 (left) and spectral+modulated combined as per Sect. 5 (right). Colored regions represent the distribution areas; the blue lines represent the prior representation

## Modulated and spectral combined analysis

From the regularities and relations discussed in Sect. 4.2, we can build new Likelihood factors from the amplitudes set $\varvec{\mathcal {A}}$ of the data DFT harmonics, normally distributed ($\mathcal {N}$):

11 $$\begin{aligned}&\mathcal {L}(\varvec{\mathcal {A}},\varvec{\mu },\textrm{Var}) \nonumber \\&\quad =\prod _{i=1}^N \mathcal {N}(\mathcal {A}_i \,|\,\mu _i,\textrm{Var}=V_0+v\cdot f(\sigma ^\textrm{Cu})S) \end{aligned}$$

12 $$\begin{aligned}&\mu _i = {\left\{ \begin{array}{ll} \mu _0 &{} i\ne N\\ \mu _0+f(\sigma ^\textrm{Cu})S &{} i=N \end{array}\right. } \,, \end{aligned}$$

where Var is given by Eq. (10), $\mu _0$ is the mean of the central harmonics, and *S* is the signal (same as explicit in Eq. (5)). The product goes for the index $i=\left\{ 1,2,4,8,17,34,68\right\}$ ($N=68$ is the last harmonic, where *S* appears in the mean $\mu _N$). The factor *f* is the fraction of the signal in the subset used, either ROI or BKG. The value of this fraction is a function of the standard deviation of the signal normal distribution, i.e., $\sigma ^\textrm{Cu}$ (same as explicit in Eq. (5)):

13 $$\begin{aligned}&f^\textrm{ROI}(\sigma ^\textrm{Cu}) =\textrm{cdf}(150\,|\,\sigma ^\textrm{Cu}) - \textrm{cdf}(-150\,|\,\sigma ^\textrm{Cu})\nonumber \\&\quad = \textrm{erf}\left( \frac{150}{\sqrt{2}\sigma ^\textrm{Cu}}\right) - \textrm{erf}\left( \frac{-150}{\sqrt{2}\sigma ^\textrm{Cu}}\right) \end{aligned}$$

14 $$\begin{aligned}&f^\textrm{BKG}(\sigma ^\textrm{Cu}) = 1-f^\textrm{ROI}(\sigma ^\textrm{Cu}) \,, \end{aligned}$$

where cdf is the Cumulative Density Function and the erf the “error function.” Since the distribution is centered on $E^\textrm{Cu}_\textrm{PEPV}$ with a standard deviation of $\sigma ^\textrm{Cu}$, the fraction in ROI tests the neighborhood width for this region, i.e., $\pm 150$ eV. The BKG region is trivially complementary to 1.

The most important *i*-th element for the signal is in the last one, i.e., $\mathcal {L}(\mathcal {A}_N,\mu _N,\textrm{Var})$. However, using the distributions of all the central harmonic sets strong constraints on the $\mu _0$ and $V_0$ parameters.

The posterior distribution is built by the product of the Likelihood factors as in equation 15:

15 $$\begin{aligned}&p(\varvec{\theta }\,|\,\varvec{\mathcal {D}}^{woc},\varvec{\mathcal {D}}^{wc},\varvec{\mathcal {A}}^\textrm{ROI},\varvec{\mathcal {A}}^\textrm{BKG})\nonumber \\&\quad = \frac{\mathcal {L}(\varvec{\mathcal {D}}^{woc},\varvec{\mathcal {D}}^{wc},\varvec{\mathcal {A}}^\textrm{ROI},\varvec{\mathcal {A}}^\textrm{BKG}\,|\,\varvec{\theta }) \cdot p(\varvec{\theta }) }{\int \textrm{d}\varvec{\theta }\, \mathcal {L}(\varvec{\mathcal {D}}^{woc},\varvec{\mathcal {D}}^{wc},\varvec{\mathcal {A}}^\textrm{ROI},\varvec{\mathcal {A}}^\textrm{BKG}\,|\,\varvec{\theta }) \cdot p(\varvec{\theta }) }\,, \end{aligned}$$

where

16 $$\begin{aligned}&\mathcal {L}(\varvec{\mathcal {D}}^{woc},\varvec{\mathcal {D}}^{wc},\varvec{\mathcal {A}}^\textrm{ROI},\varvec{\mathcal {A}}^\textrm{BKG}\,|\,\varvec{\theta }) \nonumber \\&\quad =\mathcal {L}^{woc}(\varvec{\mathcal {D}}^{woc}\,|\,\varvec{\theta })\cdot \mathcal {L}^{wc}(\varvec{\mathcal {D}}^{wc}\,|\,\varvec{\theta })\,\cdot \nonumber \\&\qquad \cdot \mathcal {L}^\textrm{ROI}(\varvec{\mathcal {A}}^\textrm{ROI}\,|\,\varvec{\theta })\cdot \mathcal {L}^\textrm{BKG}(\varvec{\mathcal {A}}^\textrm{BKG}\,|\,\varvec{\theta }) \end{aligned}$$

is the total Likelihood (factors are written in a more compact version). $\mathcal {L}^{woc}$ and $\mathcal {L}^{wc}$ are the factors in Eqs. (2) and (4), respectively; $\mathcal {L}^\textrm{ROI}$ and $\mathcal {L}^\textrm{BKG}$ are as per Eq. (11) with *f*, respectively, as per Eqs. (13) and (14).

The parameter vector $\varvec{\theta }$ contains, besides the components discussed in Sect. 3, also the new parameters introduced by Eqs. (11) and (12): $V_0^\textrm{ROI}$, $V_0^\textrm{BKG}$, $\mu _0^\textrm{ROI}$, $\mu _0^\textrm{BKG}$, $v^\textrm{ROI}$, and $v^\textrm{BKG}$, extending the dimension of the parameter space from 14 to 20. Since *v*s are estimated using the synthetic data sets (see Sect. 4.2 and the bottom plot in Fig. 5), their priors are normally distributed, centered around the respective fitted values with their uncertainties as standard deviations. The priors of the $V_0$s are uniformly distributed since the 0-signal case is unknown *a priori*. Instead, the ones from $\mu _0$s are normally distributed with parameters equal to the average and standard deviation of the central harmonics distributions since they are independent of the signal. The signal *S* and the $\sigma ^\textrm{Cu}$ (used to calculate the *f* fraction) are shared among $\mathcal {L}^{wc}$, $\mathcal {L}^\textrm{ROI}$, and $\mathcal {L}^\textrm{BKG}$.

The result of this Bayesian analysis further constraint the *S* parameter as shown in Fig. 6: on the left the marginalized posterior obtained with the Sect. 3 analysis, on the right introducing this analysis. Still, no significant signal is found, but its upper limit at 90% of C.L. is now $\bar{S}_\text {comb} = 21$. Compared to the spectral analysis described in Sect. 3, it is reduced by about $(\bar{S}_\text {spec}-\bar{S}_\text {comb})/\bar{S}_\text {spec} = 32x\%$. $\bar{S}_\text {comb}$ corresponds to the upper limits of

17 $$\begin{aligned} \left. \frac{\beta ^2}{2}\;\right| _\text {comb} < 8.50\cdot 10^{-31}&\qquad \text {(scattering)} \end{aligned}$$

18 $$\begin{aligned} \left. \frac{\beta ^2}{2}\;\right| _\text {comb} < 6.74\cdot 10^{-43}&\qquad \text {(close encounters)}\,, \end{aligned}$$

improved with respect to the sole spectral analysis (equation 9).

## Conclusions and discussions

The analysis proposed in this work uses the combined spectra (as per standard VIP-2 analysis) and takes advantage of the modulated current data-taking campaign of about 2 months as a test run. With the Discrete Fourier Transformation of the data and a thorough study of its harmonics behavior, we obtained an improved sensitivity on the probability $\beta ^2/2$ of PEP violation for electrons. No significant Pauli Exclusion Principle Violation is found. However, the proposed method improves the $\beta ^2/2$ upper limit at 90% C.L. by almost a factor of 1.5 compared to the only spectrum analysis.

From [20] (83 consecutive days with current on and 80 without it), the found upper limit on $\beta ^2/2$ was $6.8\cdot 10^{-43}$ for the close encounters case ($8.6\cdot 10^{-31}$ for the scattering). This new analysis approach yielded the same results with less than half of the data acquisition time.

The modulated data taking allows a more powerful and stringent analysis. This work is the pathfinder for future endeavors, such as future VIP-2 campaigns and the planned VIP-3.

## Figures and Tables

**Fig. 7:**
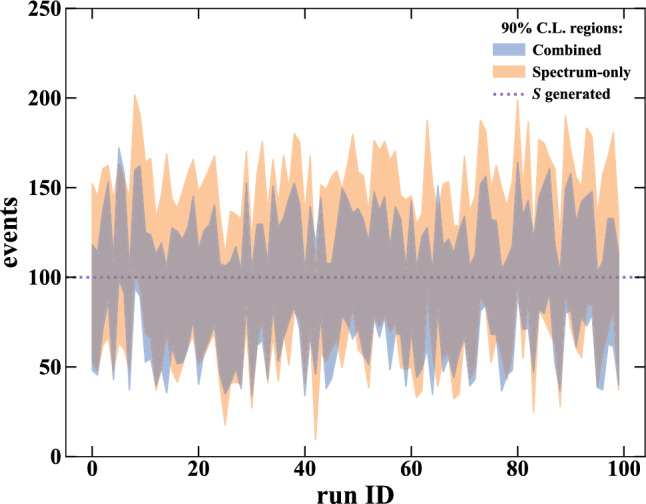
90% C.L. regions obtained for 100 toy Monte Carlo (see Appendix A), using the Combined method discussed in Sect. 5 (blue area) or Spectrum-only analysis as per Sect. 3 (orange area) in comparison. The simulated signal is 100 events in 68 days (purple dotted line), with 5000 events in each *wc* and *woc* sets

## Data Availability

This manuscript has no associated data or the data will not be deposited. [Authors’ comment: Data will be made available on reasonable request.]

## Result stability

We used the functions in Eqs. (3) and (5) with the optimized parameters $\varvec{\theta }$ from the posteriors of Eq. (15) to create a toy Monte Carlo. It simulates the random energy distribution for *woc* and *wc* *toy* sets in the 7270–8300 eV range. The *wc* is generated with $S=0$. The data are less of a few counts than 5000 events each in 68 days. Therefore, we simulated 5000 events for each toy set to be closer to the studied case.

To have the modulated toy data set with a period of $T=100$ s, we split the *wc* and *woc* toy sets into $68\cdot 24\cdot 3600/100 = 58752$ bins each. The toy data populate the bins randomly following the Poissonian statistics with an expected rate of $5000/58{,}752\simeq 0.085$. The final simulation consists of 117,504 bins alternating *wc* and *woc* events from the simulated toy sets (the first bin has *wc* elements).

Finally, we simulated 100 signal events (i.e., 1% of the total simulated events), with the energy normally distributed around $E^\textrm{Cu}_\textrm{PEPV} = 7746.73$ eV with the standard deviation of the optimized *wc* $\hat{\sigma }^\textrm{Cu}$. They are randomly spread, uniformly, across the 58752 *wc* bins.

We simulated 100 toy data sets: the run ID in Fig. 7. Using the combined method discussed in Sect. 5, i.e., both spectral and modulated current analysis, we obtained the blue regions shown in figure. For comparison, we also show in orange the results using only the spectral analysis (Sect. 3). The regions successfully and stably retrieve the generated events. The combined case has smaller regions, confirming the improvement delivered by this method even in the case of a small signal (the average reduction is $\sim 29\%$ in this case).

Displacements observed in the figure, e.g., the orange area seemingly shifted up, are not significant. The upper and lower edge differences to the reference line (average upper edge$\,-\,100$ and $100\,-\,$average lower edge, respectively) are smaller than their standard deviation: $14.587 \pm 26.680$ for the spectral analysis alone (orange) and $-10.068 \pm 24.870$ for the combined case (blue).

## Declarations

### Code availability

The code utilized in this study is available on request from the corresponding author. [Authors’ comment: Code/software will be made available on reasonable request.]

### Editorial Policies for:

Springer journals and proceedings: https://www.springer.com/gp/editorial-policies.

## References

[1] G. Luders, B. Zumino, Connection between Spin and Statistics. Phys. Rev. **110**, 1450–1453 (1958). 10.1103/PhysRev.110.1450 10.1103/PhysRev.110.1450

[2] Lieb, E.H.: Quantum mechanics, the stability of matter and quantum electrodynamics. In: Jahresbericht of the German Mathematical Society (2003)

[3] R.P. Feynman, R.B. Leighton, M. Sands, *The Feynman Lectures on Physics* (Addison-Wesley, Boston, 1963)

[4] H.S. Green, A Generalized method of field quantization. Phys. Rev. **90**, 270–273 (1953). 10.1103/PhysRev.90.270 10.1103/PhysRev.90.270

[5] Ignatiev, A.Y., Kuzmin, V.A.: Is small violation of the Pauli Principle Possible? IC/87/13 (1987)

[6] Greenberg, O.W., Mohapatra, R.N.: Local Quantum Field Theory of Violation of the Pauli Principle. Phys. Rev. Lett. 59, 2507 (1987). 10.1103/PhysRevLett.59.2507 . [Erratum: Phys.Rev.Lett. 61, 1432, 1988] 10.1103/PhysRevLett.59.2507 10035570

[7] A. Addazi, R. Bernabei, Tests of Pauli exclusion principle violations from Non-commutative quantum gravity. Int. J. Mod. Phys. A **35**(32), 2042001 (2020). 10.1142/S0217751X20420014. arXiv:1901.00390 [hep-ph] 10.1142/S0217751X20420014

[8] A.M.L. Messiah, O.W. Greenberg, Symmetrization postulate and its experimental foundation. Phys. Rev. **136**, 248–267 (1964). 10.1103/PhysRev.136.B248 10.1103/PhysRev.136.B248

[9] K. Piscicchia, Strongest atomic physics bounds on noncommutative quantum gravity models. Phys. Rev. Lett. **129**(13), 131301 (2022). 10.1103/PhysRevLett.129.131301. arXiv:2209.00074 [hep-th] 36206433 10.1103/PhysRevLett.129.131301

[10] K. Piscicchia, Experimental test of noncommutative quantum gravity by VIP-2 Lead. Phys. Rev. D **107**(2), 026002 (2023). 10.1103/PhysRevD.107.026002. arXiv:2212.04669 [hep-th] 10.1103/PhysRevD.107.026002

[11] E. Ramberg, G.A. Snow, A New Experimental Limit on Small Violation of the Pauli Principle. Phys. Lett. B **238**, 438–441 (1990). 10.1016/0370-2693(90)91762-Z 10.1016/0370-2693(90)91762-Z

[12] K. Piscicchia et al., VIP-2 —High-Sensitivity Tests on the Pauli Exclusion Principle for Electrons. Entropy **22**(11), 1195 (2020). 10.3390/e22111195 33286963 10.3390/e22111195 PMC7711554

[13] Elliott, S.R., LaRoque, B.H., Gehman, V.M., Kidd, M.F., Chen, M.: An Improved Limit on Pauli-Exclusion-Principle Forbidden Atomic Transitions. Found. Phys. 42, 1015–1030 (2012). 10.1007/s10701-012-9643-y. arXiv:1107.3118 [nucl-ex]

[14] Curceanu Petrascu, C., et al.: Experimental tests of quantum mechanics – Pauli exclusion principle violation (the VIP experiment) and future perspective. J. Phys. Conf. Ser. **306**, 012036 (2011). 10.1088/1742-6596/306/1/012036

[15] E. Milotti et al., On the Importance of Electron Diffusion in a Bulk-Matter Test of the Pauli Exclusion Principle. Entropy **20**(7), 515 (2018). 10.3390/e20070515 33265605 10.3390/e20070515 PMC7513035

[16] E. Milotti et al., Semi-Analytical Monte Carlo Method to Simulate the Signal of the VIP-2 Experiment. Symmetry **13**(1), 6 (2020). 10.3390/sym13010006 10.3390/sym13010006

[17] L. De Paolis et al., The key role of the Silicon Drift Detectors in testing the Pauli Exclusion Principle for electrons: the VIP-2 experiment. J. Phys: Conf. Ser. **1548**(1), 012033 (2020). 10.1088/1742-6596/1548/1/012033 10.1088/1742-6596/1548/1/012033

[18] De Paolis, L., et al.: Search for a signature of Pauli exclusion principle violation by VIP-2. Phys. Script. **97**, 084001 (2022). 10.1088/1402-4896/ac76ec

[19] Thompson, A.C., Vaughan, D., et al.: X-Ray Data Booklet. Lawrence Berkeley National Laboratory, University of California, California (2009). https://xdb.lbl.gov/xdb-new.pdf

[20] F. Napolitano et al., Testing the Pauli Exclusion Principle with the VIP-2 Experiment. Symmetry **14**(5), 893 (2022). 10.3390/sym14050893 10.3390/sym14050893

[21] L. Tierney, Markov Chains for Exploring Posterior Distributions. Ann. Stat. **22**(4), 1701–1728 (1994). 10.1214/aos/1176325750 10.1214/aos/1176325750

[22] W.K. Hastings, Monte Carlo sampling methods using Markov chains and their applications. Biometrika **57**(1), 97–109 (1970). 10.1093/biomet/57.1.97. https://academic.oup.com/biomet/article-pdf/57/1/97/23940249/57-1-97.pdf

[23] G. Strang, Wavelets. Am. Sci. **82**(3), 250–255 (1994)

